# Collaborative emergency preparedness and response to cross-institutional outbreaks of multidrug-resistant organisms: a scenario-based approach in two regions of the Netherlands

**DOI:** 10.1186/s12889-018-6376-7

**Published:** 2019-01-11

**Authors:** Marion de Vries, Patrick Kenis, Marleen Kraaij-Dirkzwager, Elis Joost Ruitenberg, Jörg Raab, Aura Timen

**Affiliations:** 1 Centre for Infectious Disease Control, National Institute for Public Health and the Environment (RIVM), Bilthoven, The Netherlands; 2 Centre of Environmental Safety and Security, National Institute for Public Health and the Environment (RIVM), Bilthoven, The Netherlands; 3 Tilburg Institute of Governance, Tilburg University, Tilburg, The Netherlands; 4 Athena Institute for Innovative and Transdisciplinary Research in Health Sciences, VU University Amsterdam, Amsterdam, The Netherlands; 5 Department of Organization Studies, Tilburg University, Tilburg, The Netherlands

**Keywords:** Outbreak management, Outbreak response, Network analysis, Antimicrobial resistance, Multidrug-resistant organisms

## Abstract

**Background:**

The likelihood of large-scale outbreaks of multidrug-resistant organisms (MDRO) is growing. MDRO outbreaks can affect a wide range of healthcare institutions. Control of such outbreaks requires structured collaboration between professionals from all involved healthcare institutions, but guidelines for cross-institutional procedures are, however, often missing. Literature indicates that such multi-actor collaboration is most promising when effective network brokers are present, and when the collaborative actors have clarity about the different roles and responsibilities in the outbreak response network, including collaborative structures and coordination roles. Studying these factors in an imaginary MDRO outbreak scenario, we gained insights into the expectations that health professionals in the Netherlands have in regard to the procedures required to best respond to any future cross-institutional MDRO outbreaks.

**Methods:**

For exploration purpose, a focus group discussion with ten healthcare professionals was held. Subsequently, an online-survey was conducted among 56 healthcare professionals in two Dutch regions. The survey data was analysed using social network analyses (clique analysis and centrality analysis), which provided insights into the collaborative structures and potential brokers in the outbreak response networks. Additionally, respondents were asked which healthcare institutions and which professions they would prefer as coordinating actors in the collaborative network.

**Results:**

Our results show a relatively high level of perceived clarity about the roles and responsibilities that healthcare professionals have during a joint outbreak response. The regional outbreak response networks which were studied appeared inclusive and integrated, with many overlapping groups of fully-connected healthcare actors. Social network analyses resulted in the identification of several central actors from different healthcare institutions with the potential to take on a brokerage role in the collaboration. Actors in the outbreak response networks also showed to prefer several healthcare professionals to take on the coordination roles.

**Conclusion:**

Expected collaborative structures during an imaginary regional MDRO outbreak response are relatively dense and integrated. In regard to the coordination of an MDRO outbreak response, based on both the network analysis results and the preferred coordination roles, our findings support a governance structure with several healthcare institutions involved in responding to future cross-institutional MDRO outbreaks.

**Electronic supplementary material:**

The online version of this article (10.1186/s12889-018-6376-7) contains supplementary material, which is available to authorized users.

## Background

The emergence and spread of multidrug-resistant organisms (MDRO) constitutes a major threat to global public health [1]. In the Netherlands, an outbreak in 2011 resulted in the spread of a MDRO (a multi resistant *Klebsiella pneumoniae*) to 115 hospital patients, at least three of whom died as a direct result of the infection [2]. Outbreaks of MDRO do not stay confined to the walls of individual healthcare institutions. The high level of mobility of colonised patients between healthcare settings makes cross-institutional outbreaks realistic current and future scenarios (see for example [3]). Despite the MDRO outbreak of 2011 being seen as a wake-up call, guidelines for cross-institutional MDRO outbreaks have not yet been developed in the Netherlands.

Cross-institutional outbreaks demand a multi-sectoral preparedness and response that is challenging for healthcare institutions. Previous studies have exposed weaknesses in cross-institutional preparedness and response to infectious disease outbreaks in the Netherlands [4–7]. Among these weaknesses were limited collaboration between curative institutions, public health institutions and private organisations [5, 7], late involvement of stakeholders in the response [6], and a lack of clarity regarding roles, responsibilities and mutual expectations [4, 5].

Literature indicates that effective functioning of complex multi-sectoral networks (from now on referred to as organizational networks) is more likely if the following conditions are met: First, there is a need for clarity and transparency in roles and responsibilities among the network participants. These roles and responsibilities most importantly apply to collaborative structures and coordination roles in the organizational network [8–11]. Second, in line with the need for clarity in collaborative structures and coordination roles, it is important to identify those network participants with the potential to play a brokerage or mediating role in the network [12–15]. Network brokers have the potential to create an inclusive collaborative network and can facilitate the coordination in a network.

By assessing the expectations of relevant healthcare professionals from various healthcare institutions in two regions in the Netherlands, we aim to provide an exploration of cross-institutional preparedness and response to MDRO outbreaks in the Netherlands. In the context of this paper we focus on the abovementioned factors which are important for the functioning of organisational networks. We explored the expected (and perceived clarity of) collaborative structures and preferred coordination roles of healthcare professionals, and the potential of healthcare professionals to fulfil a brokering role within the network.

## Methods

The study was conducted between November 2015 and July 2016. A mixed methodology (both in data collection and data analysis) was applied. First, a focus group discussion with healthcare professionals was held to identify outbreak response activities and healthcare professionals likely to be involved in MDRO outbreak response. Second, an on-line survey among healthcare professionals in two Dutch regions was conducted to assess expectations about the response to a cross-institutional MDRO outbreak.

As a case study for our research, we developed a MDRO outbreak scenario. A scenario was needed for the (survey) respondents to be able to reflect on the expectations they had of the response to a cross-institutional MDRO outbreak. The outbreak scenario was developed based on the report of a real MDRO outbreak in the Netherlands [16]. The scenario described a multidrug resistant *Klebsiella pneumoniae* bacterium which spread among patients in various settings (a hospital, nursing home, and private home situations). In order to adapt the scenario to the varying institutional protocols correctly, we studied current Dutch institutional MDRO guidelines for hospitals, nursing homes and home care institutions [17–19]. These documents were searched for institutional protocols concerning MDRO outbreaks (i.e. case history enquiry) and possible processes in cross-institutional MDRO outbreaks (i.e. contact investigation) relevant to our scenario. The relevant protocols and processes were integrated in the scenario. The complete MDRO outbreak scenario can be found in the Additional file 1.

### Focus group discussion

In preparation for the survey, a focus group discussion with various healthcare professionals was organised. By using a focus group discussion we aimed to a) validate the MDRO outbreak scenario, b) explore the joint (cross-institutional) response activities needed in the response to the MDRO outbreak scenario, and c) identify the healthcare professionals who were likely to be involved in these joint response activities. The exploration of joint response activities was important to provide a solid context for questions in the survey about collaboration and coordination roles. The identification of ‘likely to be involved’ healthcare professionals was an essential starting point in studying the response networks, but was also important for determining which healthcare professionals were eligible for participation in the survey.

The focus group discussion was held at the National Institute for Public Health and the Environment (RIVM) and lasted for two hours. After gaining the consent of the respondents, the discussion was audio recorded. The moderators (1st, 3rd and 6th author of this paper) presented the outbreak scenario in parts and asked respondents after each part what they thought of the scenario, what they thought should happen in terms of response activities, and who would be involved in these activities.

A group of ten clinicians and public health professionals from various healthcare settings joined the focus group discussion. The group consisted of a medical microbiologist working in a hospital and laboratory, a medical microbiologist employed in research, two infection prevention specialists working in various healthcare settings, an infectious disease control specialist employed at a regional public health service (GGD), a general practitioner (GP), an infectiologist, a geriatric specialist and two MDRO specialised policy officers employed at the RIVM. With this selection, we aimed to include professionals with varying expertise from all relevant healthcare settings, in order to gain an inclusive exploration of cross-institutional MDRO outbreak responses.

The audio-recording of the focus group discussion was transcribed and a summary including the mentioned adaptations to the scenario, joint response activities, and identified healthcare professionals, was sent for a member-check to the focus group participants.

### Online survey

An online survey was developed, piloted among five healthcare professionals, and adapted accordingly. Data was collected in May and June 2016 from healthcare professionals in two Dutch regions. The survey explored expectations towards collaboration and coordination during the outbreak response.

#### Online survey: Study sample and data collection

Survey respondents were selected based on the results of the focus group discussion (see Table 2 for the selection of respondents). The professionals who were expected to have an important role in the collaborative outbreak response were selected as desired respondents. For each healthcare institution, a maximum of three professions were selected as eligible respondents, if possible both managers and medical professionals.

In the Netherlands, 25 Regional Health Services (GGD) are responsible for the execution of regional outbreak control measures and contingency planning. Data for this study was collected in two neighbouring GGD regions. Both regions were known to be pro-active in MDRO regional preparedness, providing a good starting point for the exploration of future regional responses to cross-institutional MDRO outbreaks. In addition, the regions differ in an important aspect, namely that Region A has an academic hospital and Region B does not. By selecting these two regions we did not expect to provide generalisable findings for all the GGD regions, nor did we aim to identify all the differences between the two regions. What we did aim to provide was a first insightful exploration of the expectations of regional responses to cross-institutional MDRO outbreaks. The names of the regions have been kept confidential to ensure the anonymity of the respondents.

Respondents were approached by email. If personal email addresses were not known, the institution’s main address was used with the request to forward the invitation to the targeted healthcare professional(s). 115 email invitations were sent, 57 to Region A and 58 to Region B.

#### Online survey: Operationalisation of concepts

Respondents were asked which of the outbreak response activities (listed in Table 1) they expected to be involved in. To investigate the collaboration in the outbreak response, the respondents were asked “to whom do you give information or advice during the outbreak response”. Respondents could select several professions from a pre-defined list (see Table 2). Moreover, to gain insight into the coordination roles, respondents were asked “who do you think should have a coordinating role in the outbreak response”. The respondents could select several institutions and professionals, again based on Table 2. Finally, to assess the clarity of these roles and responsibilities (information flows and roles), respondents were asked if they perceived the collaborative structures and coordinating roles as being clear to them (on a five-point Likert scale).

**Table 1:** Cross-institutional response activities identified in the focus group discussion

1. Participate in an Outbreak Management Team to jointly decide on cross-institutional outbreak response measures	
2. Screen ex-roommates who are no longer in the hospital (in the nursing home or at home)	
3. Implement/extend infection prevention measures in the nursing home	
4. Implement infection prevention measures in the homes of the MDRO positive patients who are at home	
5. Provide information to the MDRO positive patients who are at home	
6. Answer the questions of the general public about the outbreak	
7. Share patient data with other healthcare actors and health institutions when useful for the outbreak control	
8. Communicate to the media about the outbreak	
9. Keep track of/add to the cross-institutional case register	
10. Evaluate the cross-institutional outbreak response	
11. Inform local authories about the outbreak

**Table 2:** Healthcare professions identified in the focus group discussion who could theoretically be involved in the response to an MDRO outbreak as described in the scenario^a^

Hospital	Regional Public Health Services (GGD)	Nursing Home	Homecare	General Practitioner	Medical Microbiological Laboratory	National Institute for Public Health and the Environment (RIVM)
**1. Institution management**	**9. Institution management**	**14. Institution management**	**18. Institution management**	**21. General Practitioner**	**22. Medical microbiologist** ^**b**^	**23. RIVM**
**2. Infection prevention specialist**	**10. Infection prevention specialist**	**15. Infection prevention specialist**	**19. Nurse**	–	–	–
**3. MD, medical microbiologist**	**11. MD, infectious disease control specialist**	**16. MD, Geriatric specialist**	20. Communication professional	–	–	–
4. Infectiologist	12. Infectious disease control nurse	17. Communication professional	–	–	–	–
5. Treating medical specialist	13. Communication professional	–	–	–	–	–
6. Department management	–	–	–	–	–	–
7. Outbreak management team	–	–	–	–	–	–
8. Communication professional	–	–	–	–	–	–

^a^Professions selected for survey participation are visualised in bold

^b^In the survey analysis, the responses of the medical microbiologists from hospital and laboratory settings have been taken together as one group, because the medical microbiologists proved to be regionally organised and not necessarily devoted to one institution

#### Online survey: Analysis

Using descriptive statistics, we retrieved results in preferred coordination roles, and the perceived clarity of collaborative structures and coordination roles. The data on collaboration between healthcare actors was analysed using social network analysis techniques. These techniques have been successfully applied in previous research on infectious disease control in the Netherlands (Kraaij-Dirkzwager, M.M., et al., Improving outbreak management through network analysis, in preparation) and Australia [20] and, in research on disaster management, these methods are applied frequently [13, 21, 22].

Network analyses of collaborative structures and brokerage roles were performed using Visone (Version 2.6.2) [23]. Data on collaboration (responses to the question “to whom do you give information or advice during the outbreak response”) was aggregated for respondents in the same healthcare profession from the same institution (e.g. infection prevention specialists working at GGD and professionals in management in the hospital). As the number of respondents within each healthcare profession was not equal (see the Additional files 1 and 2), relationships were included in the analysis only if 50 % or more of the respondents from a healthcare profession indicated a relationship.

The relational data was analysed in three ways. First, collaborative structures were visualised in a network graph. Second, a clique analysis was performed to gain deeper insights into the collaborative structures. Cliques are defined as smaller groups of actors (four or more) in a network in which the actors are fully-connected to each other. By looking into the number of cliques, the number of actors within each clique, and the level of clique overlap, we can make statements about the level of integration of collaboration in an organisational network [24]. Third, brokerage positions in the collaborative network were identified, using three centrality measures, namely: degree of centrality, betweenness centrality and closeness centrality. Centrality measures are measures to determine the relative importance of an actor’s position in a network based on their relationships and the structure of the network.

Degree of centrality calculates the number of direct relationships per actor in the network, and qualifies the actors with the most direct relationships as most prominent in the network. Actors with a high degree of centrality are influential because they are “in the thick of things” [21]. However, the degree of centrality is a local centrality measure and does not take into account the entire network structure. Global centrality measures such as betweenness and closeness centrality are based on direct and indirect ties and thus take into account the entire network structure. Betweenness centrality measures the probability of being on the shortest path between any pair of actors in the network. Actors with high betweenness centrality are influential because they can influence information flows and connect different parts of the network. Closeness centrality calculates the average number of steps required for each actor in the network to reach any other actor in the network [25]. The importance of an actor is, therefore, based on how quick and direct this access is to the other actors in the network via direct and indirect ties. The scores on these centrality measures were then compared with the presence of actors in cliques in the network.

### Ethics

Informed consent to participation was provided by all respondents in this study. Focus group respondents gave verbal consent for participation and survey respondents actively entered the survey after reading the respondent information. In social network analysis, anonymity and confidentiality are significant points of attention, because social network analysis focuses on the relationships of individuals or individual institutions. To ensure anonymity and confidentiality, the relational data in this study was aggregated to professions and types of institutions; the studied regions were also anonymised to ensure confidentiality. The Clinical Expertise Centre of the National Institute for Public Health and the Environment (RIVM) reviewed the research protocol and determined that this research is not subject to the Dutch law for medical research involving human subjects (WMO) [26], and therefore concluded that it was exempt from seeking further approval from the Ethical Research Committee.

## Results

### Focus group discussion: Response activities and healthcare professionals involved

The focus group discussion resulted in eleven cross-institutional response activities, and a list of healthcare professions from various healthcare institutions who were likely to be involved in the response to the MDRO outbreak. (With “professions” we refer to both single professions, such as infectious disease specialist or medical microbiologist, and to groups of people from certain professions, such as the outbreak management team.) Table 1 lists the identified cross-institutional response activities and Table 2 lists the identified healthcare professions.

### Survey: Study sample

Healthcare professions invited to take part in the survey are visualised in bold in Table 2. Fifty-six healthcare professionals, from all the invited healthcare professions, participated in the survey (26 in Region A and 30 in Region B). In the Additonal file 2, an overview is provided of the number of respondents per healthcare profession per region.

### Survey: Collaborative structures

**Fig. 1:**
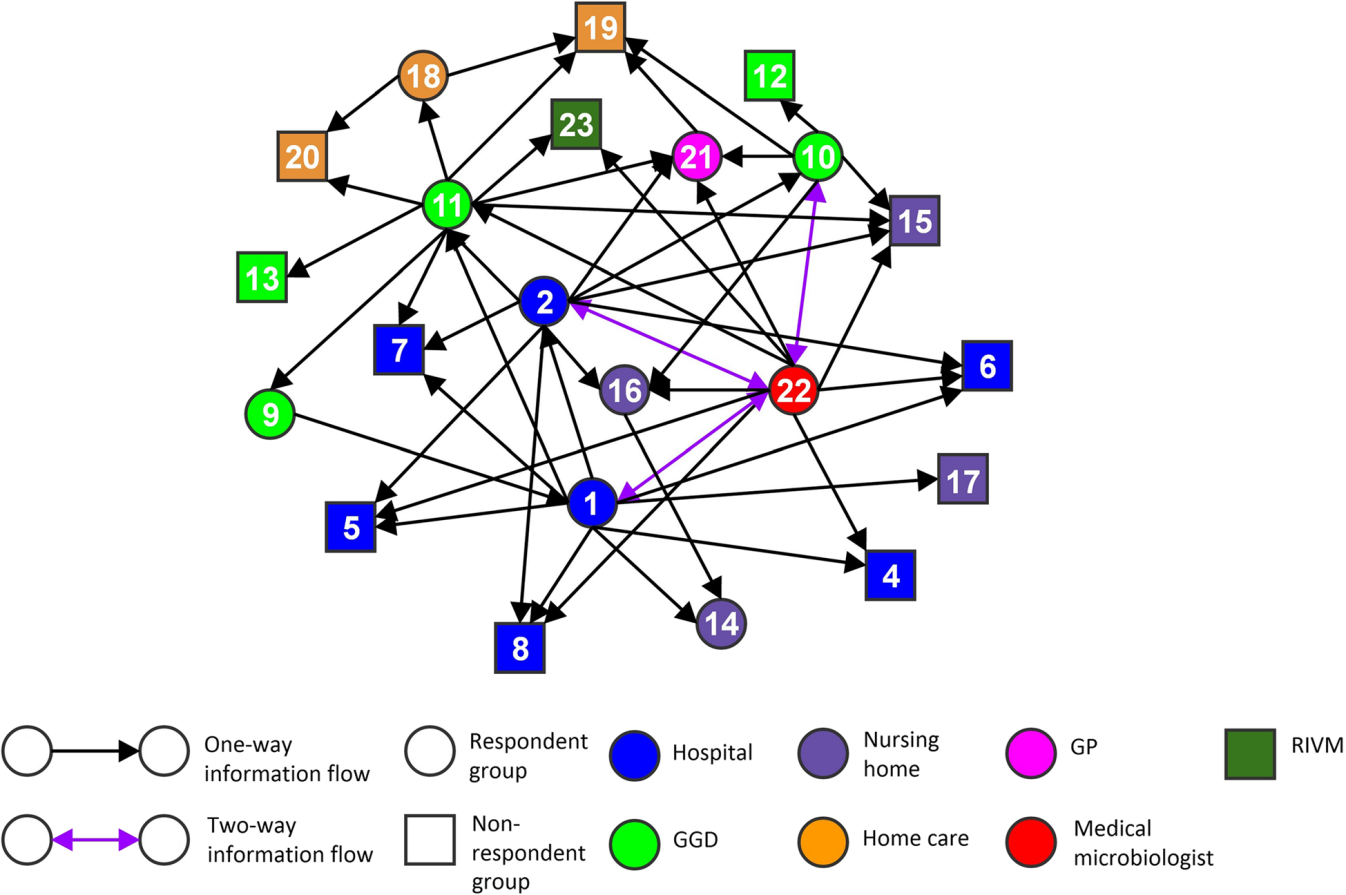
Social network visualization of indicated information flows during the outbreak in region A. Circles and squares indicate healthcare professions, subsequently healthcare professions included as respondents and healthcare professions not included as respondents. The numbers in the circles and squares correspond with the numbers of healthcare professions in Table 2 (except for number 23, which represents the RIVM as institution). The colours indicate the healthcare institutions in which the healthcare professionals operate. The direction of the arrows visualizes the information flow as indicated by the sender of the arrow. Purple arrows visualize reciprocated information flows

**Fig. 2:**
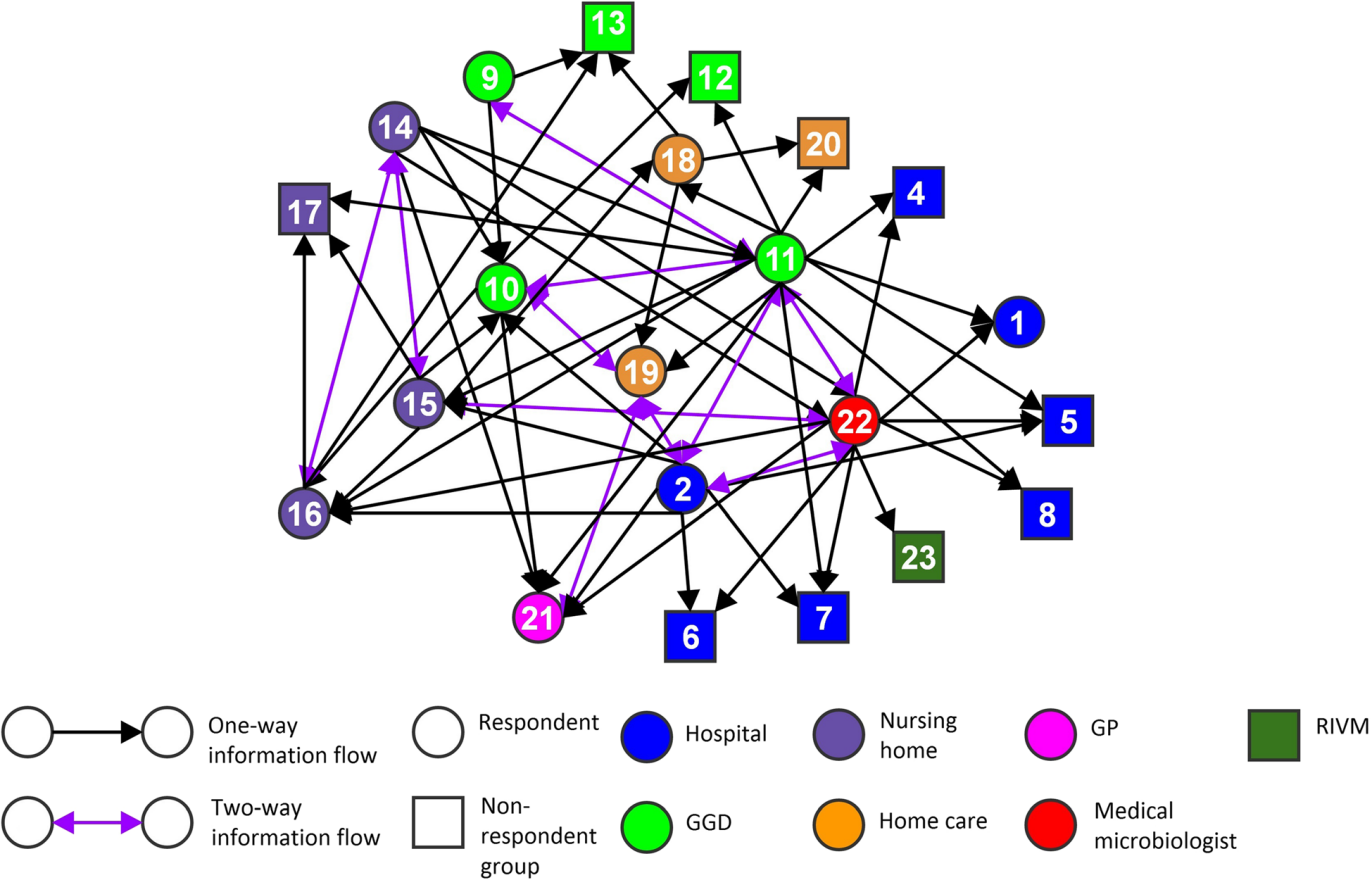
Social network visualization of indicated information flows during the outbreak in region B. Circles and squares indicate healthcare professions, subsequently healthcare professions included as respondents and healthcare professions not included as respondents. The numbers in the circles and squares correspond with the numbers of healthcare professions in Table 2 (except for number 23, which represents the RIVM as institution). The colours indicate the healthcare institutions in which the healthcare professionals operate. The direction of the arrows visualizes the information flow as indicated by the sender of the arrow. Purple arrows visualize reciprocated information flows

Network figures were constructed based on who survey respondents expected to give information or advice to during the outbreak response. The networks are shown in Fig. 1 (Region A) and Fig. 2 (Region B).

The figures show inclusive connected networks of the several healthcare professions involved in both regions. The regions differ in connectedness with Region B showing a remarkably higher amount of two-way information flows between pairs of actors than Region A. Two-way information flows imply reciprocal relationships between pairs of healthcare professions, where respondents from both health care professions indicated that they provided information to each other.

In both regions, the clique analysis shows a relatively large number of fully-connected groups of four or more healthcare professions. There are twelve cliques in Region A, each one containing four professions, and fifteen cliques in Region B, ten of which contained four professions and five with five professions. All the cliques involve healthcare professions from several healthcare institutions, and all healthcare institutions are represented in at least one clique. Tables 3 and 4 give an overview of the clique overlap by showing the representation of the different professionals in cliques, for Region A and Region B. In both regions, there are a number of professions represented in a large proportion of all the cliques in the networks. In Region A, five professions are represented in a third or more of all the cliques. In Region B, there are seven professions represented in a third or more of all the cliques. This shows that there is quite a large overlap in the cliques, which means that the collaboration is relatively integrated, and not divided into separate groups of professionals. Notable are the high representations of medical microbiologists in the cliques in Region A and GGD infectious disease control specialists in the cliques in Region B.

**Table 3:** Clique overlap in networks of information flows, Region A

Healthcare professions^a^	Presence in cliques – Proportio*n* (%)	Presence in cliques – (numbered 1–12)
Medical microbiologist	11/12 (92%)	2, 3, 4, 5, 6, 7, 8, 9, 10, 11, 12
GGD – Infection prevention specialist	7/12 (58%)	1, 2, 3, 4, 6, 7, 8
Hospital – Management	5/12 (42%)	5, 9, 10, 11, 12
GGD – Infectious disease control specialist	5/12 (42%)	1, 2, 3, 4, 5
Hospital – Infection prevention specialist	4/12 (33%)	5, 2, 9, 6
General Practitioner	3/12 (25%)	1, 2, 6
Hospital – Outbreak Management Team	2/12 (17%)	5, 11
Nursing home – Geriatric specialist	2/12 (17%)	4, 8
Nursing home – Infection prevention specialist	2/12 (17%)	3, 7

^a^Professions present in less than 10% of the cliques have not been included in the table

**Table 4:** Clique overlap in networks of information flows, Region B

Healthcare professions^a^	Presence in cliques – Proportio*n* (%)	Presence in cliques – (numbered 1–15)
GGD – Infectious disease control specialist	15/15 (100%)	1, 2, 3, 4, 5, 6, 7, 8, 9, 10, 11, 12, 13, 14, 15
Medical microbiologist	7/15 (47%)	1, 2, 3, 4, 5, 6, 7
Hospital – Infection prevention specialist	7/15 (47%)	1, 2, 3, 4, 5, 10, 11
GGD – Infection prevention specialist	6/15 (40%)	8, 9, 10, 11, 12, 13
Nursing home – Geriatric specialist	6/15 (40%)	1, 6, 9, 11, 12, 15
Nursing home – Infection prevention specialist	5/15 (33%)	1, 6, 11, 12, 15
General practitioner	5/15 (33%)	2, 7, 10, 13, 14
Nursing home – Management	4/15 (27%)	6, 7, 12, 13
Homecare – Nurse	2/15 (13%)	10, 14
GGD – Communication department	2/15 (13%)	8, 9

^a^Professions present in less than 10% of the cliques have not been included in the table

With regard to the clarity of collaborative structures, 78.5% (44/56) of the survey respondents indicated that it was clear to them who they would give information to, and 76.8% (43/56) reported that it was clear who they would receive information from. Ten point 7 % (6/56) of the respondents indicated that it was not clear to them who they would give information to, and 12.5% (7/56) declared it was not clear to them who they would receive information from.

### Survey: Coordination roles and network brokers

The coordination roles in the MDRO outbreak response networks were studied from two different angles. First, the survey respondents were asked about their preferences concerning coordination roles in the outbreak response. And second, by using centrality measures, we were able to identify the brokering actors in the outbreak response networks who are in a strategically advantageous position in the network and therefore have the potential to take on coordination roles.

#### Coordination *roles: Preference respondents*

In Tables 5 and 6 , the distribution of preferred coordinating roles is presented, respectively for Region A and Region B. On average, the survey respondents selected three institutions and/or professions (3.1 in Region A and 3.4 in Region B) as preferred coordinators. Notable is the high preference shown for the GGD to have a coordinating role in the outbreak response (selected by 16 out of 26 of the respondents in Region A and 13 out of 30 of the respondents in Region B). Also frequently selected in both regions were the RIVM, the infection prevention specialists in the GGD, hospital, and nursing home, the GGD infectious disease control specialist and the medical microbiologist. An interesting difference between the two regions is that the communication departments of GGD, hospital and nursing home were frequently indicated as a preferred coordinating role in Region B, but absent in the table of preferred coordinating roles of Region A.

**Table 5:** Respondents’ answers, in frequency and percentage, to the question “Who should, according to you, coordinate the response?” in Region A

Who should coordinate?	Frequency (total:26)	Percentage
Institutions^a^		
The GGD	16	62%
The RIVM	8	31%
The hospital	7	27%
The nursing home	4	15%
The homecare setting	3	12%
Professions^a^		
GGD – Infection prevention specialist	7	27%
Medical Microbiologist	6	23%
GGD – infectious disease control specialist	5	19%
Nursing home – Infection prevention specialist	5	19%
Hospital – Infection prevention specialist	5	19%
Nursing home – Management	3	12%
Nursing home – Geriatric specialist	3	12%

^a^Institutions and professions selected by fewer than three respondents have not been included in the table

**Table 6:** Respondents’ answers to the question “Who should, according to you, coordinate the response?” in Region B

Who should coordinate?	Frequency (total:30)	Percentage
Institutions^a^		
The GGD	13	43%
The general practitioner	4	13%
The RIVM	3	10%
The hospital	3	10%
Professions^a^		
GGD – Infectious disease control specialist	7	23%
GGD – Infection prevention specialist	7	23%
Hospital - Infection prevention specialist	7	23%
GGD – Communication department	6	20%
Medical microbiologist	6	20%
Hospital – Infection prevention specialist	5	17%
Hospital – Communication department	5	17%
Nursing home – Management	4	13%
Nursing home – Geriatric specialist	4	13%
Nursing home – Communication department	3	10%
Hospital – Outbreak Management Team	3	10%

^a^Institutions and professions selected by fewer than three respondents have not been included in the table

Despite the variety in coordinating role preferences, 75% (42/56) of the respondents indicated that it was clear to them who should on take this coordinating role; 14.2% (8/56), however, did not perceive the coordinating roles as clear.

#### Coordination roles: Network brokers

Based on centrality measures, we could identify network brokers in the organisational networks. Tables 7 and 8 show the scores for degree, betweenness and closeness centrality in percentages (100% is the total centrality in a network) for health professions in Region A and Region B. The health professions are ordered by the mean of the three centrality scores, also visualised in the tables, providing a ranking from the most central professions in the networks to the least. In addition, the proportion of cliques in which professions are represented (as also visualised in Tables 3 and 4) is added to the table for comparison. We hereby provide insights in the most influential professions in the outbreak response from a network perspective. Notable are the recurrent high centrality scores of the medical microbiologist, the GGD infectious disease control specialist, and the hospital infection prevention specialist, who show to be important network brokers when looking at both regions. There are also notable differences between the regions as the hospital management has quite higher centrality scores in Region A than in Region B, while the homecare nurse and nursing home management have significant higher centrality scores in Region B than in Region A. As expected, the professionals’ representation in cliques in the networks is in relative congruence with their centrality scores.

**Table 7:** Health professions’ scores on betweenness centrality, closeness centrality, and degree of centrality, the mean centrality, and the representation in cliques in the network in Region A

Ranking^a^	Health professions^b^	Mean centrality (%)	Betweenness centrality (%)	Closeness centrality (%)	Degree of centrality (%)	Presence in cliques proportion (%)
1	Hospital – management	17.82	26.64	16.63	10.19	5/12 (41.7%)
2	Medical microbiologist	17.60	17.91	20.08	14.81	11/12 (91.7%)
3	GGD – Infectious disease control specialist	16.63	24.30	13.54	12.04	5/12 (41.7%)
4	GGD – Infection prevention specialist	10.92	7.79	16.63	8.33	7/12 (58.3%)
5	Hospital – Infection prevention specialist	10.41	5.30	15.73	10.19	7/12 (38.3%)

^a^The ranking of the health professionals is based on the mean centrality from high to low

^b^Professions with a mean centrality lower than 10% have not been included in the table

**Table 8:** Health professions’ scores on betweenness centrality, closeness centrality, and degree centrality, the mean centrality, and the representation in cliques in the network in Region B

Ranking^a^	Health professions^b^	Mean centrality (%)	Betweenness centrality (%)	Closeness centrality (%)	Degree centrality (%)	Presence in cliques proportion (%)
1	GGD – Infectious disease control specialist	17.95	24.89	13.83	15.13	15/15 (100%)
2	Hospital – Infection prevention specialist	11.41	14.30	10.71	9.21	7/15 (46.7%)
3	Medical microbiologist	11.15	11.87	11.07	10.53	7/15 (46.7%)

^a^The ranking of the health professionals is based on the mean centrality from high to low

^b^Professionals with a mean centrality lower than 10% have not been included in the table

## Discussion

Despite the absence of specific guidelines for the response to, and preparedness for, cross-institutional MDRO outbreaks, we found quite a high perceived degree of clarity concerning the collaborative structures and coordination roles among involved healthcare professionals. In addition, based on our network analyses, we expect collaboration between various healthcare professionals to be both inclusive and densely integrated. In both regions studied, we found networks which are strongly connected by various overlapping groups of fully-connected professionals from various institutions. Finally, our results suggest that several professionals from a variety of institutions have the potential to perform a coordinating role in the outbreak response.

The high perceived degree of clarity about both coordination roles and collaboration structures is a promising result for future regional MDRO outbreak responses. Previous studies have emphasised how important it is that there is clarity in these roles and responsibilities for organisational networks in general [8–11], and for infectious disease outbreak response in particular [4, 5, 7]. Nevertheless, we should consider two points. First, cross-institutional MDRO outbreaks are relatively new in the Netherlands and policy and guidelines are still under development. It is important to realise that the fact that these roles and responsibilities have not as yet been formalised may have affected respondents view of the roles. Any ambiguity about collaboration and coordination might be more easily identified after an actual outbreak rather than by imagining the response to an outbreak scenario. A second consideration is the fact that a joint response to cross-institutional outbreaks is never clear cut. This accounts for both collaboration and coordination roles. Response networks are emergent, and their composition and structure will differ for each event [9]. Consequently, an appropriate joint response depends on many different factors (e.g. the pathogen, the scale and scope of the outbreak, the formal and informal relationships between involved individuals, and the context of each institution and each individual). In our results, we also see obvious differences between the two studied regions in network composition and centrality roles, which were also to be expected considering the different contexts, the different people involved and the limited experience with such cross-institutional outbreaks.

The dependence on context however, does not imply that having clarity about possible response procedures before a crisis is a waste of time. Guideline development is essential in preventing failures, especially in public health crises with high stakes [27]. Still, while we should adhere to guidelines to ensure good infectious disease control, these guidelines should not obstruct ad-hoc processes that respond to, and match, uncertain and fast changing situations [28, 29]. In crisis-management it has even been emphasised that ad hoc collaborations can, at times, be more important in the response to a disaster than formal structures [14]. This means that formalised procedures should be highly flexible and allow for changes and adaptation, as required by an unfolding outbreak situation.

In considering coordination roles, most respondents in our study selected several institutions and professions, who should, according to them, coordinate the outbreak response. By far, the most highly preferred coordinator was the GGD, but the RIVM, the infection prevention specialists (from the GGD, hospital and nursing home), the GGD infectious disease control specialist, and the medical microbiologist were frequently chosen too. It is not surprising that the GGD and GGD professionals are seen as potential coordinators of the MDRO outbreak response, as the GGD has a legal coordinating authority in regular infectious disease outbreaks. The RIVM however, has only an official coordinating role in infectious disease outbreaks across regions [30]. It is possible that respondents have interpreted our outbreak scenario as a potential cross-regional outbreak. Another explanation could be that respondents considered the expertise of the RIVM as needed because of the complexity of MDRO outbreaks.

Our results suggest that the GGD, the infection prevention specialists (from GGD and hospital), and the medical microbiologists, are not only preferred as coordinating professionals by many of their peers in the outbreak response, they also hold strategic positions in the collaborative networks. These findings seem to support a form of joint coordination or governance in the organisational networks, which would include (at minimum) these preferred professionals. Arguably, the GGD should have a central role in this joint governance approach.

A multi-stakeholder governance is more often considered in response to the need to improve infectious disease prevention and control [31]. In addition, this type of governance in MDRO regional preparedness and response is also congruent with recent antimicrobial resistance (AMR) policy developments in the Netherlands [32]. These policy developments are focused on the establishment of regional AMR committees of representatives from multiple healthcare institutions, which should coordinate collaboration in the joint response to cross-institutional outbreaks.

The joint type of governance supported by our results is also reflected in the modes of network governance defined by Provan and Kenis [33]. A joint committee with members from the most prominent network of participating institutions could lead and coordinate the response as a collective lead institution model composed of different actors [33]. In principle, a lead institution model of network governance is best suited for networks in which the lead institution also has responsibilities in the primary process. It is an institution which is considered by the other institutions as guiding them in a shared direction with the intrinsic motivation of working towards a collective solution. A possible complication in the case at hand is that the lead institution here is actually a group of three institutions. We, therefore, conclude that a combination of a participant governed model where participants jointly govern the network, and a lead institution model where one institution (the lead institution) governs the network and coordinates activities [33] would be the best system of governance.

A mixed or hybrid type of network governance, between a lead institution and participant governed mode, can prevent common problems occurring with coordination in networks. The leading institutions can act as brokers in collecting competencies and clarifying roles, but also have the potential of creating a sense of joint responsibility and commitment. In addition, with this type of coordination, any over-centralisation of command in response networks is prevented, which has been shown to be an undesirable condition in coordinating complex problems [14, 34].

Over-centralisation can lead to an unwillingness of network participants to collaborate, it can result in overburdening the governing party, and can inhibit ad hoc processes. Additionally in the case of over-centralisation, if the governing party is not working properly, this has huge effects on the functioning of the network as a whole. All these issues are dealt with by having an effective collective lead mode of governance. But, for such a hybrid model to work well, the conditions, the particular organisation which is in the lead, and the circumstances, should be determined before an outbreak or as early as possible at the beginning of the response.

## Limitations and opportunities for future research

Our study is - to our knowledge - the first to investigate the networks involved in a joint response to MDRO outbreaks that affect several health care institutions in the Netherlands. The findings demonstrate the value of exploring this complex, but highly relevant field for outbreak preparedness and control. The focus on a specific scenario adds to the validity of the findings, as does the iterative design and the triangulation of methods. This study also has a number of limitations, the most significant of which is the impact of the inclusion and exclusion of survey respondents in regard to the results of the social network analyses. We have controlled for the over- and under- sampling of respondents in the different target groups (by only counting the relational data confirmed by 50% of respondents from the same healthcare profession). However, our study does not include respondents from all the healthcare professions that could have a role in the response to an MDRO outbreak scenario. This means that our network analyses do not disclose the information flows between all potentially involved healthcare professions, but only show the information flows from the point of view of a selection of healthcare professionals. Nevertheless, this selection of healthcare professionals is thought to provide an adequate view of the information flows during the outbreak response, as it includes a varied group of professionals whom all were expected to have important roles in the response.

A second limitation is that our study did not investigate the exact relational data between individual healthcare professionals in a region, but instead generalised the relational data for groups of healthcare professionals. This approach ensures confidentiality, but limits the validity of the results. Finally, any generalisation of the results should be done with caution, considering the relatively small sample size, that only two regions were studied, and that the study is based on a single scenario.

Future research could provide more valuable insights by reproducing the current research with several MDRO outbreak scenarios and a larger study sample. It would also be valuable to investigate differences among regions in collaboration and coordination preferences in greater depth (by using semi-structured interviews, for example). An alternative to the social network analysis methodology which was used, and highly valuable, would be a retrospective analysis of the functioning of response networks during a real life MDRO outbreak response and/or an analysis of observed tabletop exercises. Finally, a qualitative exploration of levels of trust and prior working relationships in the networks studied would be valuable, as these elements are also considered to be highly important for the functioning of response networks [35, 36].

## Conclusion

We investigated the institutional infrastructure of a regional response to an MDRO outbreak involving several institutions in two Dutch regions. Despite the limited number of cross-institutional MDRO outbreaks which have occurred in the Netherlands to date, and the absence of specific cross-institutional guidelines, we found a relatively high perceived clarity about the roles and responsibilities among healthcare actors concerning the joint outbreak response. The regional response networks appeared quite inclusive and integrated, with many overlapping groups of fully-connected healthcare professionals. Finally, based on the preferences of healthcare professionals and the analysis of the outbreak response networks, our findings suggest that there is potential for a hybrid type of network governance, between a lead institution and participant governance mode, in line with current policy developments. We think that such an approach, if properly introduced (preferably before an outbreak occurs) will provide the basis for an effective response in a situation where we find a relatively large number of heterogeneous actors grappling with an outbreak. Further investigation to gain more insight into the institutional response to cross-institutional MDRO outbreaks would be highly valuable, given the evolving threat of antimicrobial resistance.

## Additional files

Additional file 1: The MDRO outbreak scenario. The MDRO Outbreak scenario. As a case study for our research, we developed a fictional MDRO outbreak scenario. The fictive scenario describes a multidrug resistant *Klebsiella pneumoniae* bacterium, which spread quietly among patients in various settings (a hospital, nursing home, and private home situations). (DOCX 14 kb)

Additional file 2: Overview survey respondents per healthcare profession in region A and B. The MDRO Outbreak scenario Overview survey respondents per healthcare profession in region A and B. An overview of the number of respondents participating in the survey, displayed per healthcare profession and region. (DOCX 15 kb)

## Abbreviations

GGD: The Dutch Regional Health Services (In Dutch: Gemeentelijke Gezondheidsdienst)
MDRO: Multidrug-resistant organisms
RIVM: The Dutch National Institute for Public Health and the Environment (In Dutch: Rijksinstituut voor Volksgezondheid en Milieu)
