# Supplementary material for: Collaborative emergency preparedness and response to cross-institutional outbreaks of multidrug-resistant organisms: a scenario-based approach in two regions of the Netherlands
Source: BMC Public Health. 2019 Jan 11;19:52. doi: 10.1186/s12889-018-6376-7 (PMC6329161; doi:10.1186/s12889-018-6376-7)
Supplement: Supplementary file 1 — The MDRO outbreak scenario. The MDRO Outbreak scenario. As a case study for our research, we developed a fictional MDRO outbreak scenario. The fictive scenario describes a multidrug resistant Klebsiella pneumoniae bacterium, which spread quietly among patients in various settings (a hospital, nursing home, and private home situations). (DOCX 14 kb) [file 12889_2018_6376_MOESM1_ESM.docx]

**The MDRO outbreak scenario**

An 84 year old woman (mrs. X) is admitted to the hospital on the short stay department, because of a persistent fever and low blood pressure, under the suspicion of urosepsis. The third generation cephalosporine, which she has been treated with, does not appear to work. Microbiological urine investigations reveal a NDM-1 producing *Klebsiella pneumoniae*. NDM stands for New Delhi metalobetalactamsase, an enzyme responsible for multiple resistance against antibiotics. This bacterium is resistant to co-trimoxazole and fluoroquinolones, but sensitive to aminoglycosides*,* tigecycline, and colistin. This type of resistant bacterium is highly uncommon in hospitals in the Netherlands, and its occurrence is associated with hospitalization abroad, in countries with a high prevalence of such micro-organisms.

Repeated case history enquiring about travel abroad shows that Mrs. X had recently been hospitalized with similar complaints in a hospital abroad during a holiday, but she did not mention this during her hospitalization back in the Netherlands. Upon clinical recovery, she flew back by plane, and was admitted to the hospital four days after returning to the Netherlands.

Following the guidelines, Mrs. X is treated in isolation from now on. Contact investigation is performed among her roommates (initially) and later among the whole department. The contact investigation shows colonization of other patients, and is reason to expand the investigation to all patients who could have been in contact with Mrs. Smith during her hospitalization. Some of these patients have been transferred to a nearby nursing home and some have already been discharged.

Of the 38 (ex-)patients, ten were found positive. Two of these (ex)patients are currently in the (same) nursing home. These two (ex)patients both frequently visit the hospital due to recurring complaints. Three other MDRO positive (ex)patients are currently back home. Also these (ex)patients frequently visit the hospital and are additionally daily supported by a home care institution.

This morning the regional newspaper opens with “Outbreak superbug in nursing home!”. The article dwells on the narrative of one of the patients, and discusses the growing risks of antibiotic resistance.
