# Supplementary material for: Collaborative emergency preparedness and response to cross-institutional outbreaks of multidrug-resistant organisms: a scenario-based approach in two regions of the Netherlands
Source: BMC Public Health. 2019 Jan 11;19:52. doi: 10.1186/s12889-018-6376-7 (PMC6329161; doi:10.1186/s12889-018-6376-7)
Supplement: Supplementary file 2 — Overview survey respondents per healthcare profession in region A and B. The MDRO Outbreak scenario Overview survey respondents per healthcare profession in region A and B. An overview of the number of respondents participating in the survey, displayed per healthcare profession and region. (DOCX 15 kb) [file 12889_2018_6376_MOESM2_ESM.docx]

|  | **Region A** | **Region B** | **Total** |
| --- | --- | --- | --- |
| **GGD – infectious disease control specialist** | 1 | 2 | 3 |
| **GGD - infection prevention specialist** | 1 | 1 | 2 |
| **GGD - management** | 1 | 1 | 2 |
| **General practitioner** | 4 | 4 | 8 |
| **Homecare - nurse** | 0 | 2 | 2 |
| **Homecare - management** | 2 | 1 | 3 |
| **Hospital - infection prevention specialist** | 5 | 8 | 13 |
| **Hospital – medical microbiologist** | 1 | 0 | 1 |
| **Hospital - management** | 2 | 1 | 3 |
| **Nursing home - infection prevention specialist** | 0 | 3 | 3 |
| **Nursing home - nurse** | 0 | 1 | 1 |
| **Nursing home - management** | 1 | 4 | 5 |
| **Nursing home - geriatric specialist** | 5 | 1 | 6 |
| **Laboratory - medical microbiologist** | 3 | 1 | 4 |
| **Total** | 26 | 30 | 56 |

**Overview survey respondents per healthcare profession in region A and region B**
